# Supplementary material for: Single-cell transcriptomic and genomic changes in the aging human brain
Source: bioRxiv. 2023 Nov 7:2023.11.07.566050. Preprint. [Version 1] doi: 10.1101/2023.11.07.566050 (PMC10659272; doi:10.1101/2023.11.07.566050)
Supplement: Supplement 11 [file NIHPP2023.11.07.566050V1-supplement-11.pdf]

Supplementary Figures

bioRxiv preprint doi: https://doi.org/10.1101/2023.11.07.566050; this version posted November 7, 2023. The copyright holder for this preprint (which was not certified by peer review) is the author/funder, who has granted bioRxiv a license to display the preprint in perpetuity. It is made available under aCC-BY 4.0 International license.

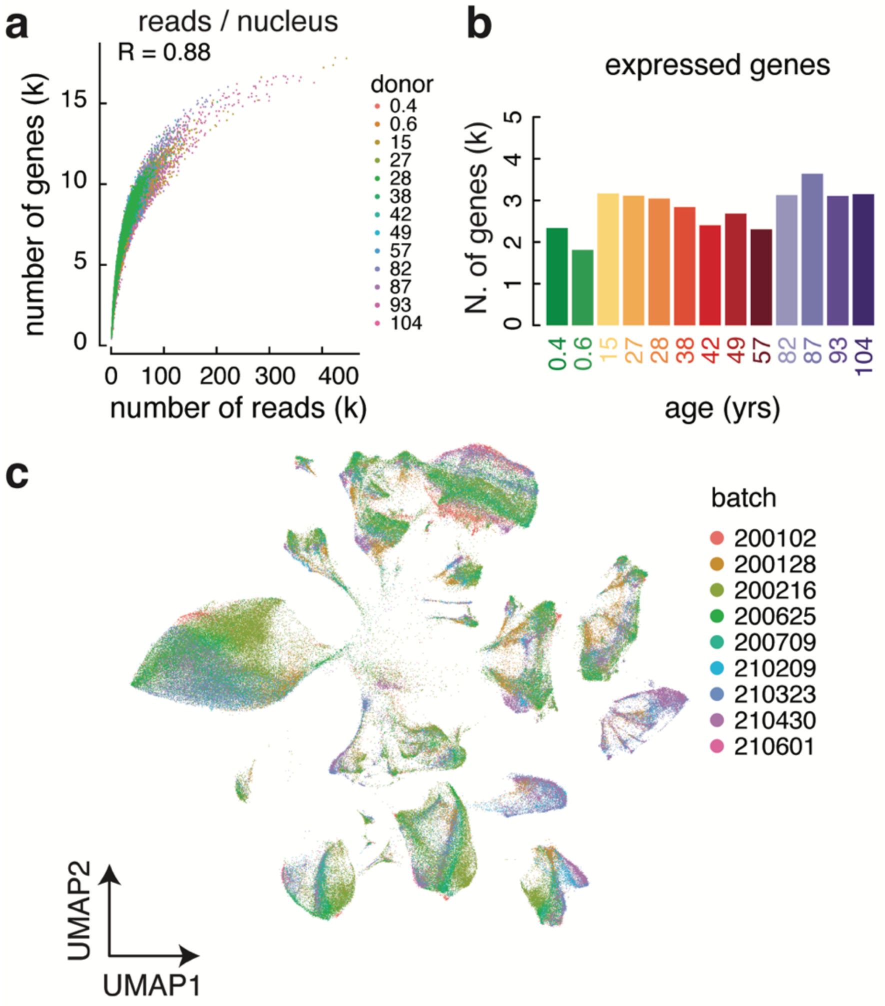

Fig. S1. Single-nucleus RNA-seq of the aging human brain. (A) Scatter plot showing the correlation between the number of expressed genes, in thousands, and the number of reads, in thousands, per nucleus, colored by donor. (B) The number of unique genes expressed in each donor did not vary significantly by age. (C) UMAP colored by preparation batch shows uniform distribution of batches across clusters.

bioRxiv preprint doi: https://doi.org/10.1101/2023.11.07.566050; this version posted November 7, 2023. The copyright holder for this preprint (which was not certified by peer review) is the author/funder, who has granted bioRxiv a license to display the preprint in perpetuity. It is made available under aCC-BY 4.0 International license.

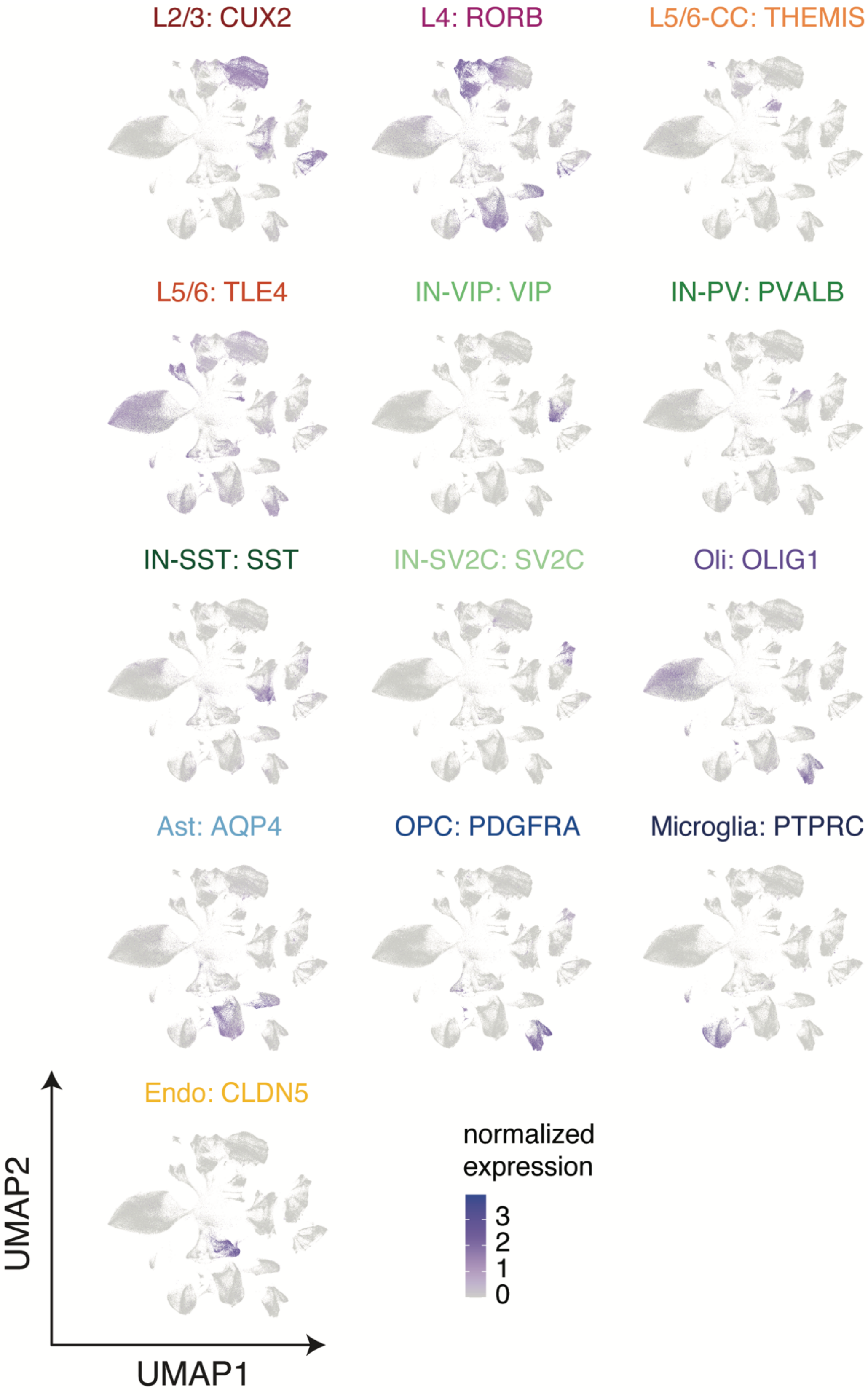

Fig. S2. Expression of Marker Genes. Expression of the canonical marker for each cell type is isolated to the corresponding cluster(s) on the UMAP demonstrating cell-type specificity of the clustering.

bioRxiv preprint doi: https://doi.org/10.1101/2023.11.07.566050; this version posted November 7, 2023. The copyright holder for this preprint (which was not certified by peer review) is the author/funder, who has granted bioRxiv a license to display the preprint in perpetuity. It is made available under aCC-BY 4.0 International license.

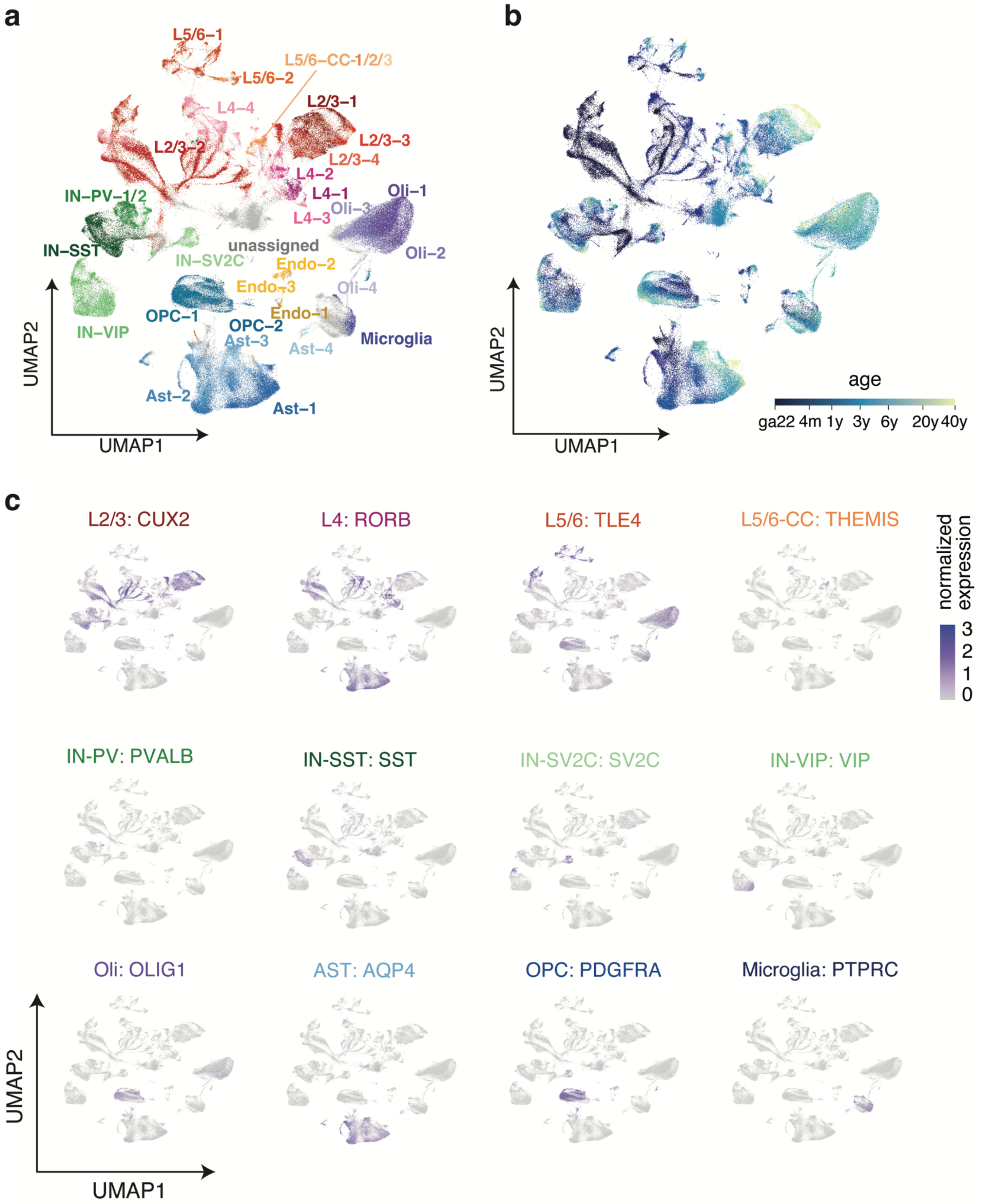

bioRxiv preprint doi: https://doi.org/10.1101/2023.11.07.566050; this version posted November 7, 2023. The copyright holder for this preprint (which was not certified by peer review) is the author/funder, who has granted bioRxiv a license to display the preprint in perpetuity. It is made available under aCC-BY 4.0 International license.

Fig. S3. Clustering of Herring et al. Data (above). (A) Donor contribution by age to each cell type. (B) Clustering of Herring et al. publicly available raw data yielded the same sub-clusters as our own data. (C) The same Herring et al. UMAP colored by age of donor reveals the same infant-specific sub-clusters for L2/3 neurons, L4 neurons, and astrocytes. Expression of the canonical marker for each cell type on the Herring et al. data is isolated to the corresponding cluster(s) on the UMAP demonstrating cell-type specificity of the clustering.

bioRxiv preprint doi: https://doi.org/10.1101/2023.11.07.566050; this version posted November 7, 2023. The copyright holder for this preprint (which was not certified by peer review) is the author/funder, who has granted bioRxiv a license to display the preprint in perpetuity. It is made available under aCC-BY 4.0 International license.

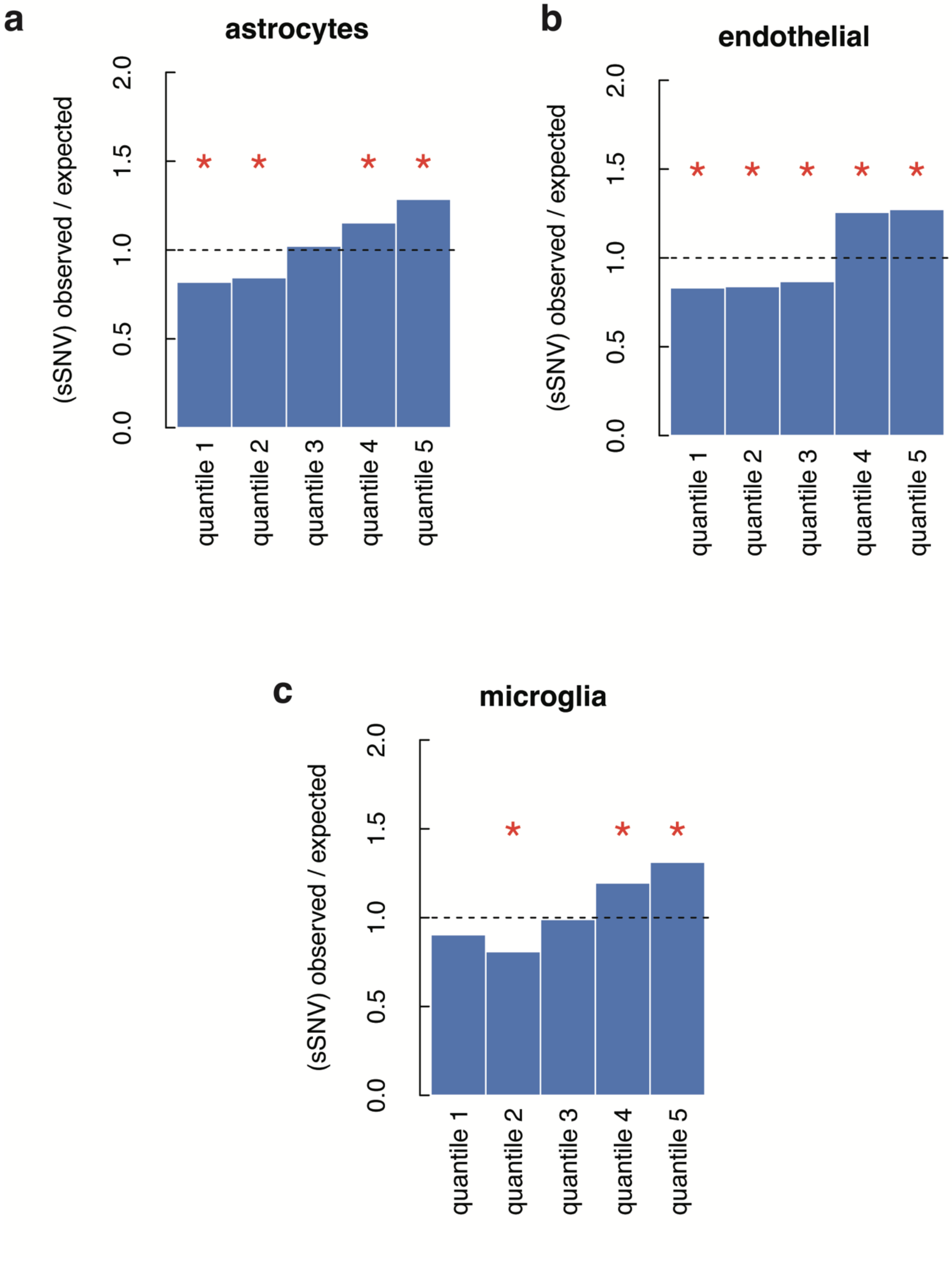

Fig. S4. Signature A1 mutation enrichment by expression level. Enrichment of Signature A1 mutations in (A) astrocytes, (B) endothelial cells, and (C) microglia correlates with gene expression. There are more Signature A1 mutations in the highest expressed genes within these cell types than expected. (x^2-test; *, $p < 0.05$).

bioRxiv preprint doi: https://doi.org/10.1101/2023.11.07.566050; this version posted November 7, 2023. The copyright holder for this preprint (which was not certified by peer review) is the author/funder, who has granted bioRxiv a license to display the preprint in perpetuity. It is made available under aCC-BY 4.0 International license.

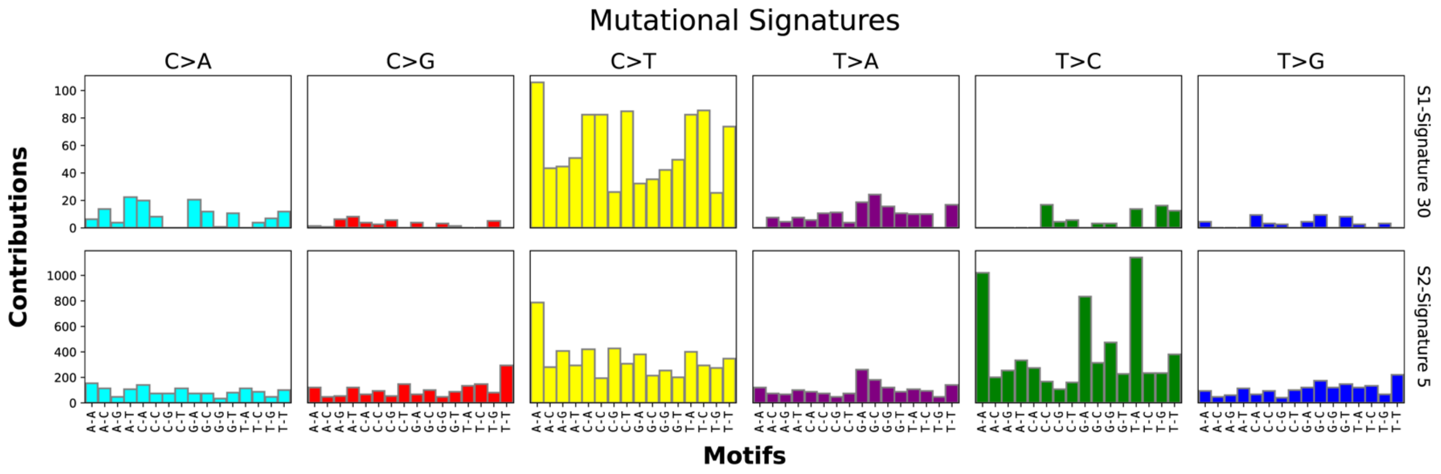

Fig. S5. De novo mutation signature analysis by python package signatureAnalyzer. Consistent with the results from MutationalPatterns, two signatures were predicted. The first signature is dominated by C>T mutations, consistent with signature A2, and the second is dominated by T>C mutations, consistent with signature A1.

bioRxiv preprint doi: https://doi.org/10.1101/2023.11.07.566050; this version posted November 7, 2023. The copyright holder for this preprint (which was not certified by peer review) is the author/funder, who has granted bioRxiv a license to display the preprint in perpetuity. It is made available under aCC-BY 4.0 International license.

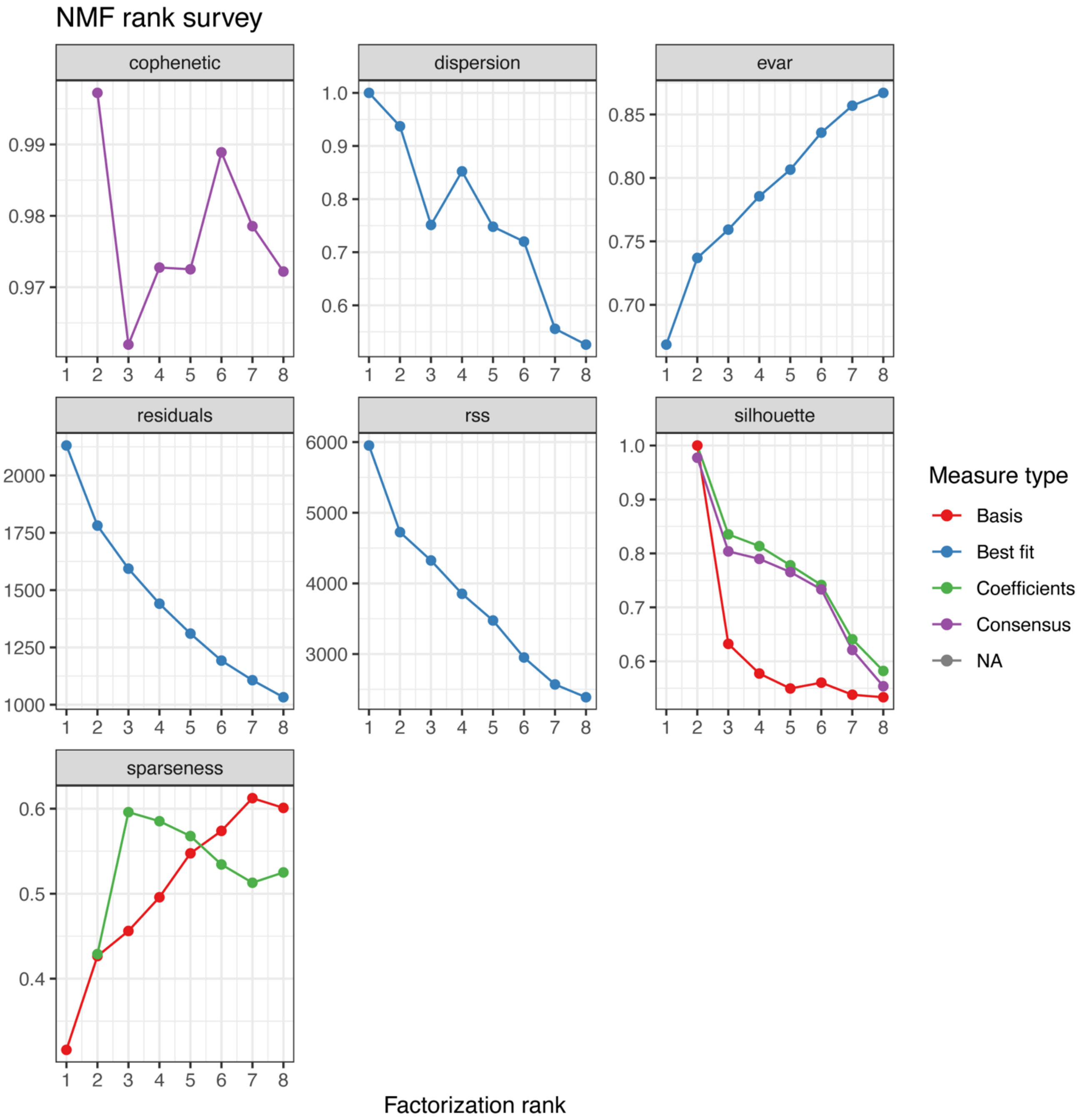

Fig. S6. Signature metrics for de novo mutation signature analysis by MutationalPatterns. Mutational signatures of sSNVs are fitted with a NMF-based framework. According to the metrics, we concluded two is the number of signatures that maximize the cophenetic of the decomposition.

bioRxiv preprint doi: https://doi.org/10.1101/2023.11.07.566050; this version posted November 7, 2023. The copyright holder for this preprint (which was not certified by peer review) is the author/funder, who has granted bioRxiv a license to display the preprint in perpetuity. It is made available under aCC-BY 4.0 International license.

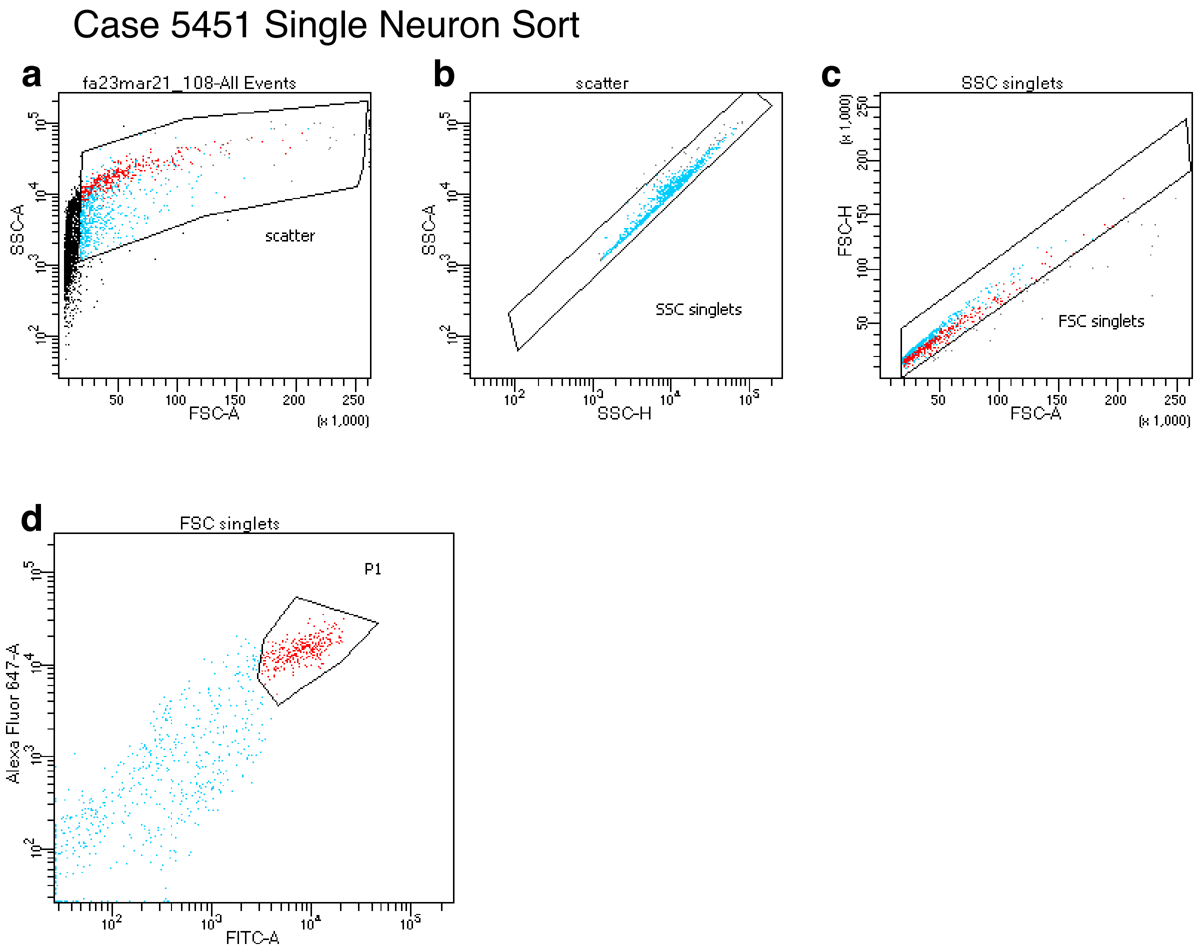

Fig. S7. Representative FACS plot for single neuron sorting. (**a**) Gating used to isolate nuclei from debris. (**b, c**) Gating used to remove doublets. (**d**) Gating used to identify neuronal nuclei.

Supplementary Table Legends

Table S1. Extended Donor Information. Case ID, sex, age at death, number of snRNA-seq nuclei, number of scWGS nuclei, post mortem interval (PMI) in hours, cause of death, RNA integrity number (RIN), years in storage, and brain bank information for each donor.

Table S2. Table of marker genes distinguishing each cluster. Gene name, $\log_2$ fold change of expression in marker cluster vs. other clusters, percent of cells in the marker cluster expressing the gene, percentage of cells in other clusters expressing the gene, p-value, q-value, and cluster identity.

Table S3. Table of cell counts in each cluster broken out by donor. Each column is a cluster and each row is a donor.

Table S4. Infant-specific differentially expressed genes. Table listing genes differentially expressed between infant-specific clusters and adult clusters of the same cell type. Negative $\log_2$ fold change indicates more expression in adult clusters. Positive $\log_2$ fold change indicates more expression in infant clusters.

Table S5. Enriched GO terms for infant specific clusters. Term name, false discovery rate (FDR), fold enrichment, number of control genes, number of differentially expressed genes number of differentially expressed genes in the term, category represented in figure 2C, and gene hits for each GO germ. Up-

bioRxiv preprint doi: https://doi.org/10.1101/2023.11.07.566050; this version posted November 7, 2023. The copyright holder for this preprint (which was not certified by peer review) is the author/funder, who has granted bioRxiv a license to display the preprint in perpetuity. It is made available under aCC-BY 4.0 International license.

regulated indicates the genes that have higher expression in the infant-specific cluster and down-regulated indicates genes that have higher expression in the adult sub-clusters of that type.

Table S6. Differentially expressed genes common to multiple classes of cells. Genes differentially expressed across cell types. All genes were down-regulated in elderly brains in all cell types. Excitatory and inhibitory neuron hits indicate the number of excitatory or inhibitory neuron cell types for which the gene was differentially expressed. Glial hits includes endothelial cells along with all glia.

Table S7. Elderly vs. adult differentially expressed genes. Table listing genes differentially expressed between elderly cases and adult cases. Negative $\log_2$ fold change indicates more expression in adult clusters. Positive $\log_2$ fold change indicates more expression in adult clusters.

Table S8. Enriched GO terms for elderly vs. adult differentially expressed genes. Term name, false discovery rate (FDR), fold enrichment, number of control genes, number of differentially expressed genes number of differentially expressed genes in the term, category represented in figure 4A, and gene hits for each GO germ. Up-regulated indicates the genes that have higher expression in elderly brains and down-regulated indicates genes that have higher expression in the adult brains.

Table S9. Sequencing statistics. Tabs show sequencing statistics for single cells and associated bulk tissues.

Table S10. Somatic single nucleotide variation calls. Summary tab provides SCAN2 sensitivity information for each single cell. Detailed information provides the annovar annotation for each SNV identified with SCAN2.
